# Letter from the Editor in Chief

**DOI:** 10.19102/icrm.2020.110203

**Published:** 2020-02-15

**Authors:** Moussa Mansour


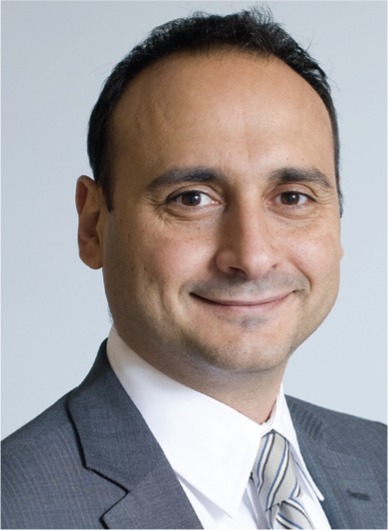


Dear Readers,

At the 25^th^ annual scientific Atrial Fibrillation (AF) Symposium held this year in Washington, DC, scientists and regulatory and health care industry leaders presented on some of their recent findings, techniques, and technologies. One session that garnered special attention was the late-breaking clinical trials session.

Chauhan et al. first presented the results of a randomized controlled trial on focal source and trigger (FaST) mapping in AF. In this investigation, FaST areas were identified using multielectrode mapping and a proprietary real-time analysis of sustained, periodic bipolar and unipolar electrograms lasting five seconds. Eighty patients with paroxysmal and persistent AF were randomized to either pulmonary vein isolation alone (PVI) or PVI in addition to FaST ablation (PVI + FaST). At 12 months of follow-up, 72% of the PVI + FaST patients were free of AF relative to 51% of patients in the PVI-alone group [hazard ratio: 1.91; 95% confidence interval (CI): 0.91–4.02; p = 0.077]. It is hoped that these findings will be followed by the conduct of a larger multicenter study.

The findings of a second study examining the value of extensive catheter linear ablation on persistent AF (CLEAR-AF) were presented by Hu et al. This prospective, randomized, multicenter trial enrolled 214 patients with persistent AF who were randomly assigned to either group 1 (PVI + left atrial roof line + left atrial anterior wall line) or group 2 (PVI + left atrial roof line). Mitral valve isthmus lines were added in both groups if the AF could not be terminated after all approaches above were adopted. Two-year follow-up revealed no significant difference was present in the sinus rhythm maintenance rate between the two groups after a single ablation procedure (60.2% versus 56.7%; p = 0.651). However, more recurrences in the extensive ablation group in the form of atrial flutters were observed, which did better following repeat ablation, than AF recurrences.

Shah et al. discussed a study examining the correlation between weight change and AF recurrence after catheter ablation. Multivariate logistic regression showed that patients who lost weight after ablation had a 71% lower risk for AF recurrence at one year when compared with patients who did not (odds ratio: 0.29; 95% CI: 0.11–0.80; p = 0.013). It is hoped that the results of this study will trigger the initiation of a larger multicenter study randomizing patients to ablation versus ablation with aggressive weight loss.

The fourth presentation, which detailed 12-month efficacy and safety results from the global prospective multicenter STOP PERSISTENT AF study, was introduced by Calkins et al. This study found that cryoballoon ablation using the Arctic Front Advance™ system (Medtronic, Minneapolis, MN, USA) is safe and effective in the ablation of persistent AF. The 12-month efficacy rate of ablation using this technology was 55%. The study confirmed the hypothesis that, to achieve higher success rates in treating persistent AF, adjunctive ablation in addition to PVI will likely be required.

Next, Reddy et al. presented data from a first-in-human trial of a lattice-tip temperature-controlled radiofrequency ablation catheter, focusing on PVI outcomes and linear lesion durability. In this study, 60 patients were treated in three European centers using this novel technology. In addition to PVI, some participants underwent tricuspid and mitral isthmus ablation. Following this study, there were two important findings: first, the rate of durable PVI at remapping study was 96.3% and, second, at 291 days ± 106 days of follow-up, the 12-month Kaplan–Meier estimate for freedom from all atrial arrhythmias was 94.4% ± 3.2%. The findings of this study have been published in *JACC: Clinical Electrophysiology*.^1^

The sixth presentation, also by Reddy et al., was an initial report from PersAFOne, a study examining the use of pulsed-field ablation to treat persistent AF with PVI plus posterior wall ablation. As part of this investigation, 15 patients with persistent AF were enrolled in two European centers and underwent PVI and posterior left atrial wall ablation. This pilot study supports the view that pulsed-field ablation is a promising modality and facilitates ultrafast ablation of AF with no major complications. Larger multicenter randomized studies are needed to confirm the early results.

The last study was presented by Duytschaever et al. on the safety and effectiveness of PVI with standardized VISITAG SURPOINT™ workflow (Biosense Webster, Diamond Bar, CA, USA). This prospective, nonrandomized study conducted across 17 European sites enrolled 340 patients with paroxysmal AF. A novel ablation point display algorithm was used to help operators create contiguous lesions for ipsilateral PVI. Kaplan–Meier analysis estimated rates of 12-month freedom from all atrial arrhythmia recurrence of 78.3% (95% CI: 73.8%–82.8%) per stringent arrhythmia monitoring and 89.4% (95% CI: 86.0%–92.8%) per standard-of-care monitoring.

The abovementioned landmark late-breaking clinical trials are expected to have a significant impact on the field of AF ablation, with the novel energy sources and mapping techniques described improving the success rates of AF ablation and reduce complications. Further research in this regard is eagerly anticipated.

I hope that you enjoy reading this issue of the journal and find its content to be of value.

Sincerely,


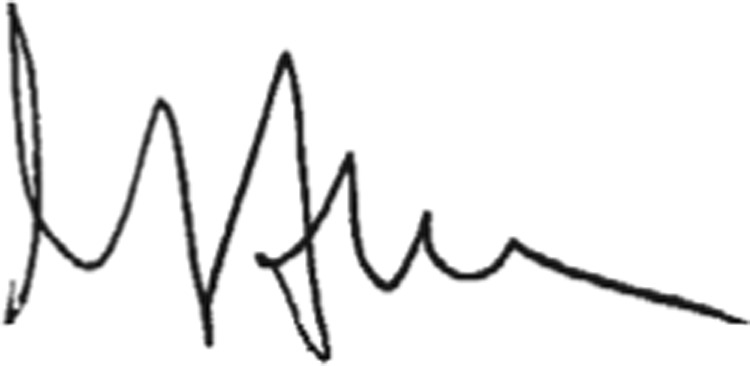


Moussa Mansour, md, fhrs, facc

Editor in Chief

The Journal of Innovations in Cardiac Rhythm Management

MMansour@InnovationsInCRM.com

Director, Atrial Fibrillation Program

Jeremy Ruskin and Dan Starks Endowed Chair in Cardiology

Massachusetts General Hospital

Boston, MA 02114

## References

[1] Reddy VY, Neužil P, Peichl P (2020 Jan 24). A lattice-tip temperature-controlled radiofrequency ablation catheter: durability of pulmonary vein isolation and linear lesion block.. JACC Clin Electrophysiol..

